# In-Depth Duodenal Transcriptome Survey in Chickens with Divergent Feed Efficiency Using RNA-Seq

**DOI:** 10.1371/journal.pone.0136765

**Published:** 2015-09-29

**Authors:** Guoqiang Yi, Jingwei Yuan, Huijuan Bi, Wei Yan, Ning Yang, Lujiang Qu

**Affiliations:** National Engineering Laboratory for Animal Breeding and MOA Key Laboratory of Animal Genetics and Breeding, College of Animal Science and Technology, China Agricultural University, Beijing, China; CSIR Institute of Genomics and Integrative Biology, INDIA

## Abstract

Since the feed cost is a major determinant of profitability in poultry industry, how to improve feed efficiency through genetic selection is an intriguing subject for breeders and producers. As a more suitable indicator assessing feed efficiency, residual feed intake (RFI) is defined as the difference between observed and expected feed intake based on maintenance and growth. However, the genetic mechanisms responsible for RFI in chickens are still less well appreciated. In this study, we investigated the duodenal transcriptome architecture of extreme RFI phenotypes in the six brown-egg dwarf hens (three per group) using RNA sequencing technology. Among all mapped reads, an average of 75.62% fell into annotated exons, 5.50% were located in introns, and the remaining 18.88% were assigned to intergenic regions. In total, we identified 41 promising candidate genes by differential expression analysis between the low and high RFI groups. Furthermore, qRT-PCR assays were designed for 10 randomly chosen genes, and nine (90.00%) were successfully validated. Functional annotation analyses revealed that these significant genes belong to several specific biological functions related to digestibility, metabolism and biosynthesis processes as well as energy homeostasis. We also predicted 253 intergenic coding transcripts, and these transcripts were mainly involved in fundamental biological regulation and metabolism processes. Our findings provided a pioneering exploration of biological basis underlying divergent RFI using RNA-Seq, which pinpoints promising candidate genes of functional relevance, is helpful to guide future breeding strategies to optimize feed efficiency and assists in improving the current gene annotation in chickens.

## Introduction

Chicken meat and egg products continue to be an important source of nutrition for most people around the world. In the past decades, many yield-related traits in chickens have been greatly improved to meet the ever-increasing global demand [1, 2]. Currently, certain traits such as daily weight gain, total egg number and age at first egg have come close to their selection limits in nature due to long-term artificial selection. Meanwhile, feed prices would likely contribute to a substantial increase although feed has accounted for more than 60% of the total production cost [3, 4]. The increasing cost with no further increase in production kept pressure for us to investigate how to improve feed efficiency. In this sense, breeding more efficient chickens would mean big savings and enhance the profitability for breeders and producers.

Two major assessment criteria for feed efficiency are feed conversion ratio (FCR) and residual feed intake (RFI), respectively. FCR is defined as the amount of feed consumed per unit of egg weight for layers, and is not a desirable measurement for several statistical and biological reasons [4–6]. Thus, an alternative concept RFI was proposed and calculated as the difference between observed feed intake and the expected feed requirement based on maintenance and growth [5, 7, 8]. RFI may be a more suitable strategy evaluating feed efficiency due to its phenotypic independence in relation to growth and production traits used in its estimation [3]. It should be noted that RFI shows moderate to high heritability, indicating that genetic improvement could be accelerated by exploring associated genes and markers to be used in molecular breeding. Furthermore, several previous studies demonstrated that selection for low RFI (superior feed efficiency) may lead to lower the production cost and environmental nitrogen pollution in chickens and other livestock [9–11]. Therefore, pursuing the potential functional genes and genetic markers underlying RFI is an intriguing issue.

Currently, several previous studies have unveiled some candidate quantitative trait loci (QTL) involved in RFI through association and linkage analyses [12–15], but these genetic evidence is still not enough. Furthermore, all these work started from the genome-scale perspective. Considering that divergent RFI performances should result from different expression levels of related genes, so monitoring the transcriptome changes in chickens with extreme RFI would offer a new opportunity to decipher its underlying mechanisms. In recent years, RNA sequencing (RNA-Seq) technology has emerged as a powerful and revolutionary approach to quantify gene expression levels and survey detailed transcriptome profiling at unprecedented resolution and sensitivity [16, 17]. Compared with microarray platform, RNA-Seq has several clear advantages such as a wider dynamic range of expression levels, higher accuracy and reproducibility, lower background noise and ability to detect novel transcripts [16, 18, 19]. Moreover, RNA-Seq method has attracted considerable interest and received great success concerning many economic traits in livestock [20–24]. Hence, applying RNA-Seq to dig out involved functional genes would serve as a great complement to traditional genomic methods.

In order to identify causal genes modulating RFI performance and get a closer insight in transcriptome architecture in chickens, we conducted a global transcriptome profiling including differential expression analysis, novel transcript prediction and functional annotation based on high-quality RNA-Seq data from duodenal epithelial tissues. Our findings will allow a better understanding of the underlying mechanisms implicated in RFI, contribute to breeding more efficient chickens by genetic improvement and help to optimize the current chicken gene model.

## Materials and Methods

### Ethics statement

The whole protocols and procedures involving animals were performed in accordance with *the Guidelines for the Care and Use of Experimental Animals* established by the Ministry of Agriculture of China (Beijing, China). All animal work was approved by the Animal Welfare Committee of China Agricultural University (permit number: SYXK 2007–0023). Before tissue sampling, birds were humanely sacrificed by cervical dislocation. All efforts were made to minimize their suffering.

### Sample selection and tissue harvest

A pure line of brown-egg dwarf layers (DW), maintained and selected mainly for egg production for over 10 years in the Poultry Genetic Resource and Breeding Experimental Unit of China Agricultural University [25], was used in this study. At 28 wk of age, a total of 252 hens were randomly selected and transferred to individual cages with intelligent system for recording individual feed intake (FI) and egg mass production (EM). These hens were kept under the 16L:8D light regimen and raised in the same environment with feed and water *ad libitum*. The FI and EM were measured at two independent stages in which the first one was from 32 to 44 wk of age and the second was from 57 to 60 wk of age. The individual body weight (BW) was surveyed at the start and end of each stage to calculate the mean BW (MBW), metabolic BW (MBW^0.75^) and daily BW gain (BWG). The residual feed intake (RFI) index was estimated with the model as follows:

$$\text{RFI}=\text{FI}-\left({\text{b}}_{0}+{\text{b}}_{1}{\text{MBW}}^{0.75}+{\text{b}}_{2}\text{BWG}+{\text{b}}_{3}\text{EMD}\right)$$

where RFI = residual feed intake, FI = daily feed intake, MBW^0.75^ = metabolic body weight, BWG = daily body weight gain, EMD = daily egg mass (adjusted for abnormal eggs), b_0_ = the intercept, and b_1_, b_2_, b_3_ = partial regression coefficients. The RFI estimates for each stage were calculated with the linear model fit function (lm) implemented in R.

We preferred those samples with extreme RFI phenotypes in a consistent pattern at two experimental stages, considering that the desired birds should show stable performances in both the early and late periods. The average RFI rank in two stages was used to prioritize samples because the mean was subject to outliers or extreme values. At the end of the whole experimental period (61 wk of age), we selected six samples consisting of two groups (three biological replicates per group) to represent two distinct RFI performances. In particular, besides that FI and RFI were significantly lower in the low RFI group, almost all phenotypes in both groups were similar. Table 1 details the measurements of RFI and its component traits at the two stages. The heritability estimates of RFI in this population are close to 0.30, though the larger sample size should be required to increase the reliability. For RNA isolation, duodenal epithelial tissues as a major part of the digestive system were harvested immediately from postmortem samples, frozen in liquid nitrogen and then stored at -80°C until further processing.

**Table 1:** Descriptive statistics of feed efficiency and relevant traits.

Trait	First stage (32 to 44 wk of age)		Second stage (57 to 60 wk of age)	
	Low	High	Low	High
**FI**	84.42 ± 8.77	99.92 ± 4.46	88.36 ± 6.88	120.56 ± 7.11
**MBW**	1336.43 ± 193.30	1270.10 ± 112.71	1394.42 ± 147.62	1401.03 ± 192.30
**BWG**	1.69 ± 0.79	1.20 ± 0.58	0.38 ± 0.94	0.89 ± 2.03
**EMD**	40.59 ± 4.06	43.27 ± 0.28	42.54 ± 3.03	42.80 ± 2.92
**RFI**	-7.83 ± 0.79	7.98 ± 2.77	-11.77 ± 0.66	19.20 ± 4.13
**FCR**	2.08 ± 0.01	2.31 ± 0.10	2.01 ± 0.03	2.72 ± 0.03

Abbreviations: FI = daily feed intake; MBW = mean body weight; BWG = daily body weight gain; EMD = daily egg mass; RFI = residual feed intake; FCR = feed conversion ratio.

### RNA extraction, library preparation and sequencing

Total RNA was isolated using TRIzol reagent (Invitrogen, USA) after grinding the frozen duodenal sample into fine powder under liquid nitrogen environment. The quality and quantity of RNA were monitored by 1% agarose gels, NanoPhotometer spectrophotometer (Implen, CA, USA) and Qubit 2.0 Flurometer (Life Technologies, CA, USA). For the eligible samples, the RNA integrity number (RIN, a score from 0 to 10) was accessed using Agilent Bioanalyzer 2100 system (Agilent Technologies, CA, USA). Only RNA samples with RIN larger than seven were used for cDNA library construction. For each sample, library with about 200 bp insert size was prepared with TruSeq RNA Sample Prep Kit v2 (Illumina, San Diego, CA, USA), and then was subjected to 2 × 100 bp paired-end (PE100) sequencing on a HiSeq 2000 instrument (Illumina). All six samples were sequenced on one lane. The raw sequence data from this article is publicly available in the NCBI Short Reads Archive (SRA) with accession number SRP055561 (BioProject number: PRJNA276492). The experiment accessions for the six chickens are SRX892810-SRX892815.

### Differential expression analysis

For ensuring high-quality data, we removed low-quality reads and reads containing adapter contamination or at least 10 Ns from raw data (FASTQ format) using in-house Perl scripts. Prior to downstream analyses, the overall quality of clean data was further examined using FastQC v0.11.2 (http://www.bioinformatics.babraham.ac.uk/projects/fastqc/). The Galgal4 reference assembly (FASTA format) and annotated gene model (GTF format) were downloaded from Ensembl database (ftp://ftp.ensembl.org/pub/release-76/). For each library, we estimated the actual insert size distribution after indexing the reference genome using the Bowtie2 v2.2.3 with default parameters [26]. After that, the mean read insert size and corresponding standard deviation (SD) as well as 10 maximum multiple hits (*—max-multihits* = 10) were used for TopHat2 v2.0.12, to improve the accuracy of reads mapping and expression analysis [27, 28]. All other parameters were set to the default values. The distribution of mapped reads over exons, introns and intergenic regions was determined using the BEDTools suite [29].

Based on resulting alignment and Ensembl annotation files, gene-level read counts were enumerated using HTSeq v0.6.1 Python tool with the default “union” mode [30]. To enhance the statistical power for identifying differentially expressed genes (DEGs), we removed those genes with weak expression levels using the HTSFilter package [31]. The DESeq2 package [32] was employed to distinguish DEGs between the low and high RFI groups. DESeq2 first used empirical Bayes shrinkage method to estimate dispersions and fold changes by modeling read counts as following a Negative Binominal distribution. And then the Wald test *P*-value was inferred to evaluate the statistical significance. The derived *P*-values were adjusted for multiple testing using Benjamini-Hochberg method [33], in order to control the false discovery rate (FDR) due to numerous tested genes in a typical RNA-Seq dataset. Finally, the DEGs were declared at a significant level of |log_2_ (fold change)| > 0.585, raw *P*-value < 0.01 and FDR < 0.05. After that, we downloaded the latest chicken QTL database (http://www.animalgenome.org/cgi-bin/QTLdb/GG/index, Release 26) [34], and compared putative DEGs with the reported QTLs associated with feed efficiency traits (FI, FCR and RFI). It should be note that the three traits should share similar genetic basis due to the strong genetic correlations among them.

### Quantitative RT-PCR confirmations

To confirm our differential expression results, we conducted quantitative reverse transcription PCR (qRT-PCR) assays for 10 randomly selected DEGs in the same RNA samples used for RNA-Seq. The total RNA was used for first-strand cDNA synthesis using EasyScript cDNA Synthesis Super Mix kit (TransGen Biotech, Beijing, China) according to standard procedures. The full cDNA sequence for each gene was downloaded from NCBI database, and corresponding primers were designed using Primer5.0 software. Prior to qRT-PCR validation, we accessed the primer quality using an 8-point standard curve in triplicate to ensure the similar amplification efficiencies between target and control primers. All qRT-PCR reactions were conducted in triplicate on the ABI Prism 7500 sequence detection system (Applied Biosystems group) using SYBR green chemistry. The thermal cycle conditions were as follows: 1 cycle of pre-incubation at 50°C for 2 min and 95°C for 10 min, 40 cycles of amplification (95°C for 10 s and 60°C for 1 min). Relative gene expressions of DEGs were calculated using the 2^-ΔCt^ method, with the housekeeping gene *GAPDH* serving as internal control. To compare with the sequencing-based results, we converted the mean 2^-ΔCt^ value for each group to fold change by dividing it by the mean value for the control. For evaluating the concordance between predicted and observed expression levels of DEGs, regression analysis was conducted with the linear model fit function (lm) implemented in R.

### Prediction and characterization of intergenic transcripts

To investigate and classify the novel transcript patterns, we performed a global transcriptome profiling. The aligned reads were assembled into transcripts based on reference-guided assembly strategy implemented in Cufflinks suite v2.2.1 [35, 36]. The resulting individual annotation file was compared with the Ensembl annotation model using the *Cuffcompare* option to capture both native and novel transcripts. For analyzing those unknown intergenic transcripts (“u”-labeled transcripts), *Cuffmerge* was first used to merge the assemblies from all samples. And then the merged transcript assembly was regard as input for *Cuffdiff2* to estimate transcript abundances. Due to the potential presence of the assembly artifacts, unspliced pre-mRNAs and possible DNA contamination, we only kept transcripts with total length between 200 bp and 20 kb and harboring at least two exons. In addition, all transcripts with fragments per kilobase of exon per million mapped reads (FPKM) > 1 and its 95% confidence interval lower boundary > 0 were included to eliminate lowly expressed transcripts which were generally considered to be transcriptional noise. Finally, only transcripts located at least 500 bp away from any known genes remained considering that these sequences might be extended exons of known genes. The sequences of eligible transcripts were subsequently extracted by *gffread* and fed to Coding Potential Calculator (CPC) to predict their coding potential [37]. To ensure high level of reliability, the transcript with CPC score > 1 was classified as protein-coding candidate, and < -1 was non-coding.

### Functional enrichment and annotation analyses

To gain insight into the biological functions of DEGs, the enriched Gene Ontology (GO) terms and Kyoto Encyclopedia of Genes and Genomes (KEGG) pathways were determined using GOSeq R package designed to correct for gene length bias [38]. The functional group with adjusted *P*-value < 0.05 and at least two DEGs in the background terms was considered significantly overrepresented. In addition, we performed functional annotation for these putative coding transcripts using Blast2GO v2.8.0 tool based on similarity searches and existing annotation associations [39]. These sequences were first blasted against the NCBI non-redundant database using BLASTX option with an E-value threshold of 0.001 and a maximum of 20 hits. The output in XML format was mapped to GO database and assigned to different functional categories. Subsequently, InterProScan annotation, ANNEX modification and GO-Slim reduction were conducted to refine the functional annotations. All steps were carried out at the default settings recommended by Blast2GO.

## Results

### Overall assessment for mapping statistics

**Fig 1:**
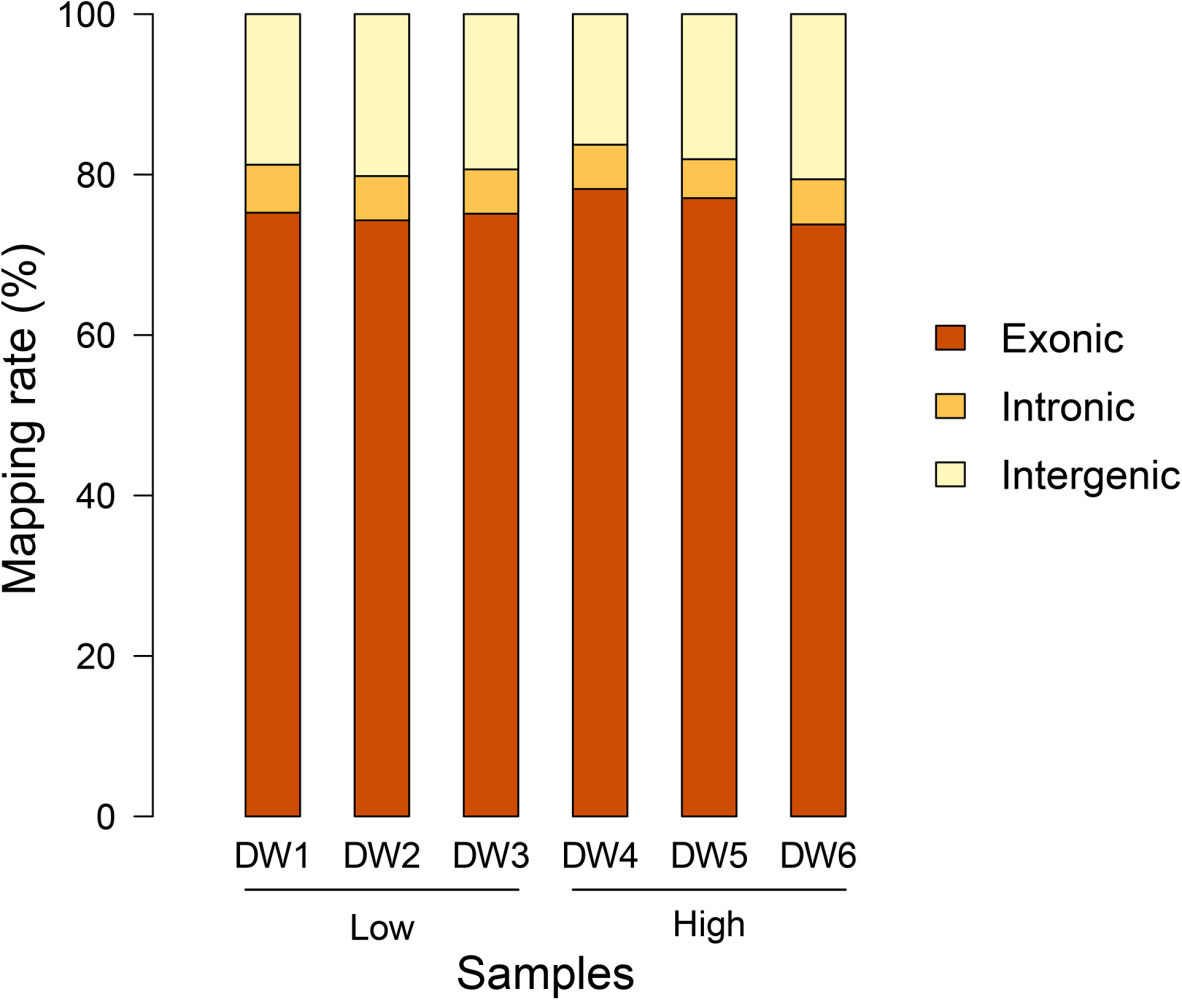
The percentage of reads mapped to exonic, intronic and intergenic regions.

The RNA-Seq of six duodenal epithelial samples yielded around 513.8 million of raw 100-bp paired-end reads. After quality filtering, each sample remained approximately 8.1 gigabases (Gb) high-quality sequence data, ranging from 7.1 to 9.5 Gb. Using TopHat2 aligner, more than 86.06% of clean reads per sample were mapped back to the Galgal4 assembly. Almost 94.08–95.88% reads were aligned in a unique manner, while 4.12–5.92% as multiple-mapped reads. The detailed information of data quality and mapping statistics is presented in Table 2. Among all mapped reads, the vast majority of which (73.79–78.20%) fell into annotated exons, 16.27–20.59% was within the large intergenic territory, and only 4.85–5.98% was located in introns (Fig 1).

**Table 2:** Summary statistics for sequence quality and alignment information of six samples.

Statistic	DW1	DW2	DW3	DW4	DW5	DW6
**Group**	Low	Low	Low	High	High	High
**Raw reads**	80,258,102	86,305,796	101,028,434	81,602,282	88,785,322	75,829,126
**Clean reads**	75,513,580	81,073,172	95,231,110	77,507,042	83,282,532	71,345,656
**Q20 (%)**	96.62	96.57	96.53	96.69	96.49	96.70
**Q30 (%)**	90.06	90.02	89.84	90.11	89.76	90.30
**GC content (%)**	49.31	48.70	48.99	48.60	48.98	48.35
**Total mapped reads**	64,983,672	70,550,893	83,756,195	67,730,446	72,506,607	62,959,041
**Unique mapped reads**	61,806,076	66,466,874	79,010,564	64,942,605	68,247,434	59,232,077
**Multiple mapped reads**	3,177,596	4,084,019	4,745,631	2,787,841	4,259,173	3,726,964
**Spliced mapped reads**	16,046,950	14,852,291	18,975,442	12,296,897	14,147,055	16,682,423
**Mapping rate (%)**	86.06	87.02	87.95	87.39	87.06	88.25
**Insert size (SD)**	200 (57)	211 (55)	201 (57)	189 (51)	196 (54)	198 (57)

Q20 and Q30 represent the proportion of bases with a Phred quality score greater than 20 and 30, respectively.

### Differential expression profiling

As a preliminary, we used HTSeq to determine the number of aligned reads per gene across all samples. According to the defined counting criterion, 75.13–77.79% of mapped reads were successfully matched to known gene model, and the remaining 22.21–24.87% were classified as “ambiguous” (reads which assigned to multiple genomic features) or “no feature” (reads which could not be assigned to any genomic feature). For enhancing the statistical power, weakly expressed genes were first filtered out according to derived Jaccard similarity index from HTSFilter package. Finally, those genes with normalized expression levels less than proposed threshold 6.473 in all six samples were removed, resulting in a total of 13,235 (77.36%) genes to be fed to DESeq2 for subsequent differential analysis.

**Fig 2:**
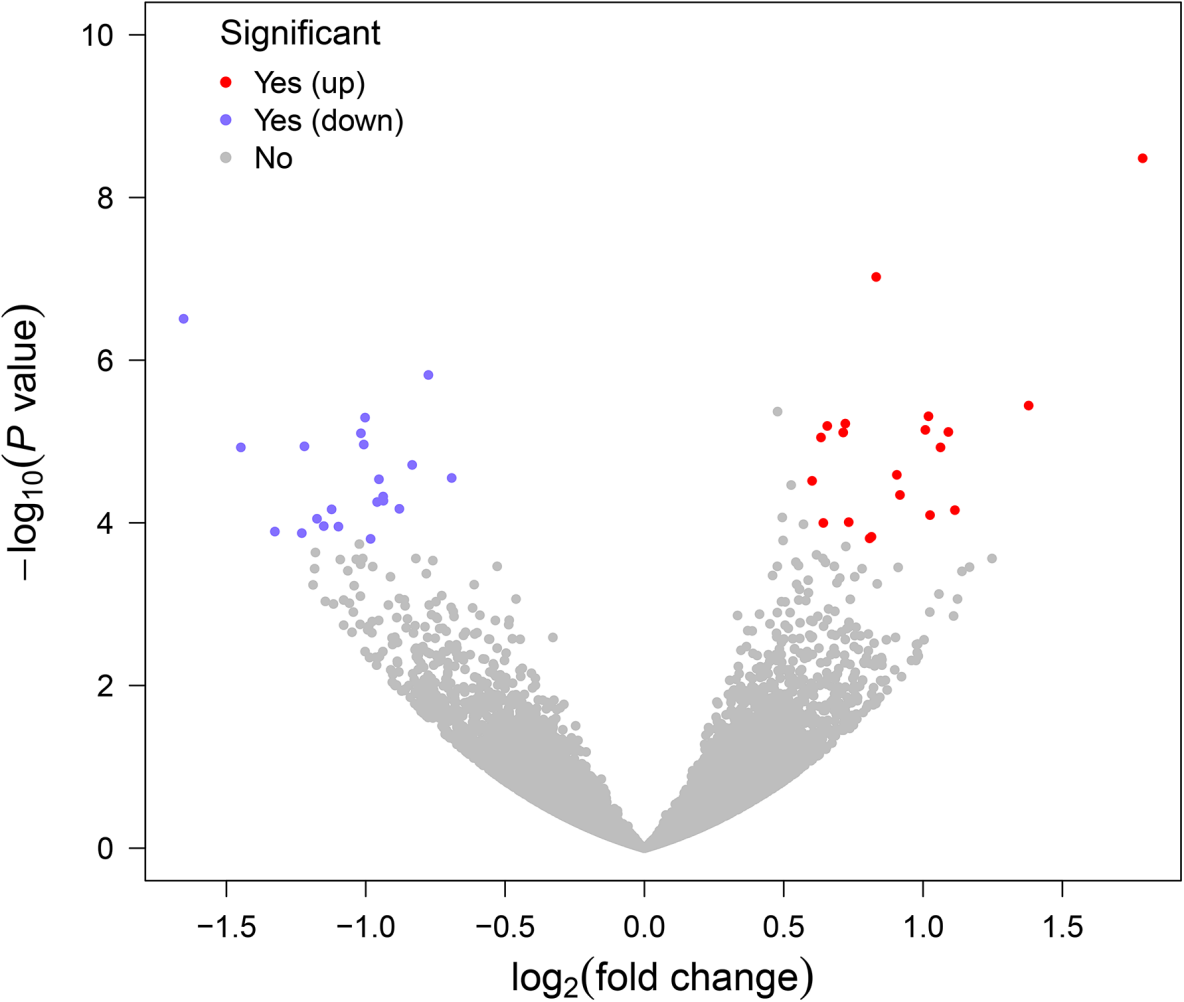
Volcano plot reporting *P* values against fold changes. The Volcano plot indicates-log_10_ (*P*-value) for genome-wide genes (Y-axis) plotted against their respective log_2_ (fold change) (X-axis). The red and blue dots represent significantly up- and down-regulated genes between the low and high residual feed intake groups respectively.

**Fig 3:**
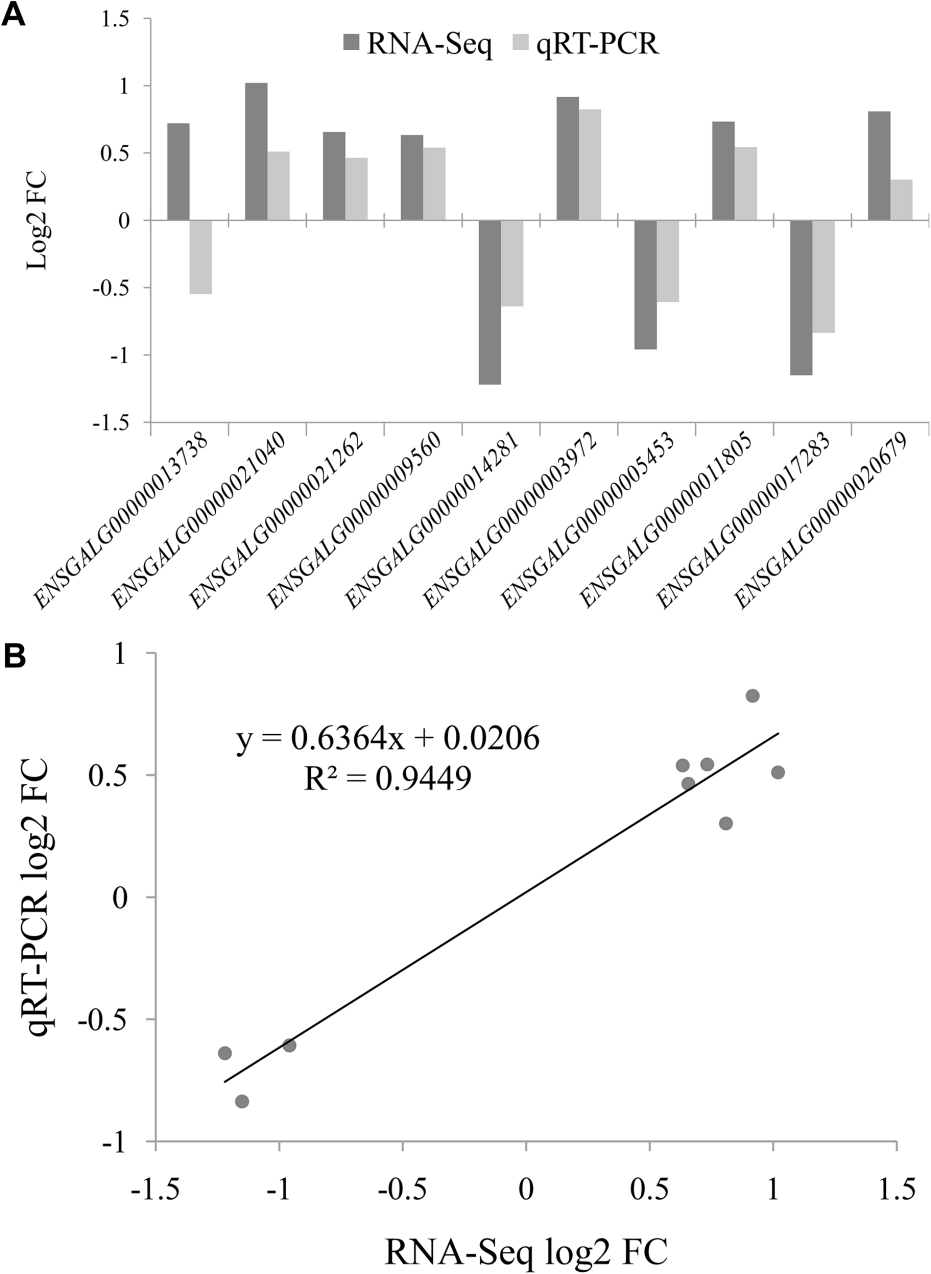
Illustrating of qRT-PCR confirmation results for 10 selected differentially expressed genes. (**A**) X-axis represents 10 selected genes for qRT-PCR assays and Y-axis represents the log_2_ (fold change) derived from RNA-Seq and qRT-PCR. (**B**) Regression analysis of the log_2_ (fold change) values between RNA-Seq and qRT-PCR.

In total, we detected 41 significant differentially expressed genes (DEGs) in response to divergent RFI based on aforementioned cutoffs. Of these putative genes, 21 were down-regulated in the low RFI group and the other 20 were up-regulated in the same group (Table 3, Fig 2). Moreover, we found that only five DEGs are located in seven previously reported QTL regions associated with feed efficiency traits (Table 3). To validate the accuracy of our predictions, 10 DEGs were randomly selected for qRT-PCR assays using the same RNA samples used for RNA-Seq. Primer sequences and validation results are listed in S1 Table. The comparative results of the fold changes predicted by RNA-Seq and qRT-PCR were displayed in Fig 3. For 10 chosen DEGs, nine showed the concordant expression patterns between RNA-Seq and qRT-PCR results (Fig 3A). After excluding the only one gene with opposite expression level, the computational and experimental fold changes in our study also showed a strong positive correlation with R^2^ = 0.9449 (Fig 3B).

**Table 3:** Detailed information of differentially expressed genes responsible for divergent RFI.

Ensembl gene ID^a^	Gene name	Chr	Log2 FC (Low / High)	*P*-value	FDR	Reported QTL (ID)
ENSGALG00000007596	*-*	12	1.79	3.30E-09	4.29E-05	
ENSGALG00000000498	*ACE*	27	0.83	9.51E-08	0.000619	
ENSGALG00000007028	*-*	4	-1.65	3.10E-07	0.001344	
ENSGALG00000005065	*PLA2G4A*	8	-0.78	1.53E-06	0.004961	
ENSGALG00000009341	*CRAMP1L*	14	-1.00	5.10E-06	0.007383	
ENSGALG00000009372	*PHC3*	9	-1.02	7.95E-06	0.007383	
ENSGALG00000013738	*OPTN*	1	0.72	6.01E-06	0.007383	
ENSGALG00000016400	*RSAD2*	3	1.09	7.61E-06	0.007383	
ENSGALG00000019211	*MAEL*	1	1.38	3.62E-06	0.007383	FCR QTL (1821)
ENSGALG00000020488	*C11orf24*	5	0.71	7.77E-06	0.007383	
ENSGALG00000021040	*HAGHL*	14	1.02	4.88E-06	0.007383	
ENSGALG00000021262	*GHITM*	6	0.66	6.44E-06	0.007383	
ENSGALG00000021274	*-*	17	1.01	7.21E-06	0.007383	
ENSGALG00000009560	*MSMO1*	4	0.63	8.90E-06	0.007719	FCR QTL (6688), RFI QTL (6689)
ENSGALG00000006558	*WDR60*	2	-1.01	1.09E-05	0.008112	FI QTL (1870)
ENSGALG00000014281	*N4BP2*	4	-1.22	1.15E-05	0.008112	
ENSGALG00000015192	*GZMM*	28	-1.45	1.19E-05	0.008112	
ENSGALG00000027166	*ART7B*	1	1.06	1.18E-05	0.008112	
ENSGALG00000002042	*KNOP1*	14	-0.83	1.94E-05	0.012594	
ENSGALG00000026299	*C3orf52*	1	0.91	2.57E-05	0.015886	
ENSGALG00000002643	*SELP*	8	-0.69	2.82E-05	0.016463	
ENSGALG00000015353	*BBX*	1	-0.95	2.91E-05	0.016463	
ENSGALG00000009006	*-*	2	0.60	3.05E-05	0.016505	
ENSGALG00000003972	*FAXDC2*	13	0.92	4.55E-05	0.022748	
ENSGALG00000005441	*NFIB*	Z	-0.94	4.76E-05	0.022906	
ENSGALG00000007996	*ANKRD44*	7	-0.94	5.33E-05	0.024778	
ENSGALG00000005453	*PLCG2*	11	-0.96	5.56E-05	0.024925	FCR QTL (6734)
ENSGALG00000003719	*CHD9*	11	-1.12	6.82E-05	0.028419	
ENSGALG00000010311	*NAV3*	1	-0.88	6.74E-05	0.028419	RFI QTL (6674), FCR QTL (6675)
ENSGALG00000021235	*NPY6R*	13	1.11	6.99E-05	0.028419	
ENSGALG00000007412	*MPZL2*	24	1.03	8.06E-05	0.031747	
ENSGALG00000006818	*KMT2A*	24	-1.17	8.89E-05	0.033025	
ENSGALG00000011227	*CDS1*	4	0.64	0.000101	0.035376	
ENSGALG00000011805	*-*	4	0.73	9.81E-05	0.035376	
ENSGALG00000009791	*PROX1*	3	-1.10	0.000111	0.036161	
ENSGALG00000017283	*CCND2*	1	-1.15	0.00011	0.036161	
ENSGALG00000017204	*SESN3*	1	-1.33	0.000128	0.040612	
ENSGALG00000013846	*KIAA1244*	3	-1.23	0.000134	0.041432	
ENSGALG00000023925	*-*	6	0.81	0.000148	0.044899	
ENSGALG00000014700	*DHX29*	Z	-0.98	0.000157	0.045423	
ENSGALG00000020679	*PLEKHS1*	6	0.81	0.000155	0.045423	

Abbreviations: Chr = chromosome; FC = fold change; FDR = false discovery rate; FCR = feed conversion ratio; RFI = residual feed intake; FI = feed intake.

^a^Identification of the gene according to Ensembl genes database 76

### Functional annotation of differential expressed genes

To investigate the associated functional categories of the 41 most significant genes, enriched GO terms and pathways were determined by the GOSeq package. It should be note that no one GO term or pathway remained statistically significant after Benjamini-Hochberg correction, likely due to incomplete gene annotation information in chickens. Therefore, we kept categories with an unadjusted threshold of *P*-values < 0.05 and at least two DEGs in the background terms to assess the potential functions. Finally, we identified 17 plausible GO terms which are mainly involved in organic acid biosynthetic process, carboxylic acid biosynthetic process, small molecule biosynthetic process, carboxylic acid metabolic process, single-organism biosynthetic process and lipid metabolic process (S2 Table). The KEGG pathway analysis revealed six overrepresented pathways, including steroid biosynthesis, p53 signaling pathway, glycerophospholipid metabolism, VEGF signaling pathway, phosphatidylinositol signaling system and metabolic pathways (S3 Table).

### Landscape of intergenic transcripts

Considering that a high percentage (18.88%) of total mapped reads were assigned to intergenic regions, identifying and characterizing these unknown transcripts would be beneficial to improve current gene model. A total of 36,513–39,527 transcripts per sample were assembled from Cufflinks software, of which 43.09–46.72% were predicted to have a complete match with the annotated intron chain, 25.27–27.68% were potentially novel isoforms of known genes and 15.67–17.21% may involve the novel intergenic transcripts. A summary about transcripts classified into different classes is shown in Table 4. To survey the architecture of intergenic-expressed regions, all six samples were merged using *Cuffmerge* command, resulting in a total of 9,796 non-redundant and novel intergenic transcripts. After strict quality assurance procedures, a total of 472 qualified transcripts were included into the downstream analyses. According to the putative CPC scores of analyzed transcripts, 38 were predicted as transcripts with no coding ability and 253 were classified as protein-coding transcripts. It should be note that a majority of coding transcripts (156 out of 253, 61.66%) were located in those unknown contigs while 38.34% were assigned to anchored chromosomes.

**Table 4:** Summary of transcripts assembled (TA) with Cufflinks in each sample.

Class code^b^	DW1^a^		DW2		DW3		DW4		DW5		DW6	
	TA	%	TA	%	TA	%	TA	%	TA	%	TA	%
**=**	16,979	46.10	17,074	44.69	17,060	46.72	17,034	43.09	17,016	45.37	16,971	45.35
**c**	4	0.01	3	0.01	3	0.01	4	0.01	5	0.01	4	0.01
**e**	751	2.04	881	2.31	751	2.06	805	2.04	739	1.97	604	1.61
**i**	1,609	4.37	2,215	5.80	1,811	4.96	3,711	9.39	2,241	5.98	2,338	6.25
**j**	10,196	27.68	9,934	26.00	9,537	26.12	9,989	25.27	10,054	26.81	10,031	26.81
**o**	393	1.07	346	0.91	343	0.94	341	0.86	363	0.97	411	1.10
**p**	972	2.64	996	2.61	881	2.41	1,044	2.64	1,025	2.73	1,009	2.70
**s**	0	0.00	0	0.00	0	0.00	2	0.01	1	0.00	1	0.00
**u**	5,771	15.67	6,575	17.21	5,951	16.30	6,391	16.17	5,899	15.73	5,884	15.72
**x**	155	0.42	179	0.47	176	0.48	206	0.52	161	0.43	169	0.45
**Total**	36,830	100.00	38,203	100.00	36,513	100.00	39,527	100.00	37,504	100.00	37,422	100.00

^a^DW1 to DW3 correspond to chickens of the low residual feed intake group, while DW4 to DW6 correspond to chickens of the high residual feed intake group

^b^Class codes described by Cuffcompare: " = " Complete match of intron chain, "c " Contained in the reference annotation, "e" Possible pre-mRNA fragment, "i " An single exon transcript falling entirely within a reference intron, "j " New isoform, "o" Unknown, generic overlap with reference, "p" Possible polymerase run-on fragment, “s” An intron of the transfrag overlaps a reference intron on the opposite strand, "u" Unknown, intergenic transcript, “x” Exonic overlap with reference on the opposite strand.

### Potential functional roles for coding transcripts

**Fig 4:**
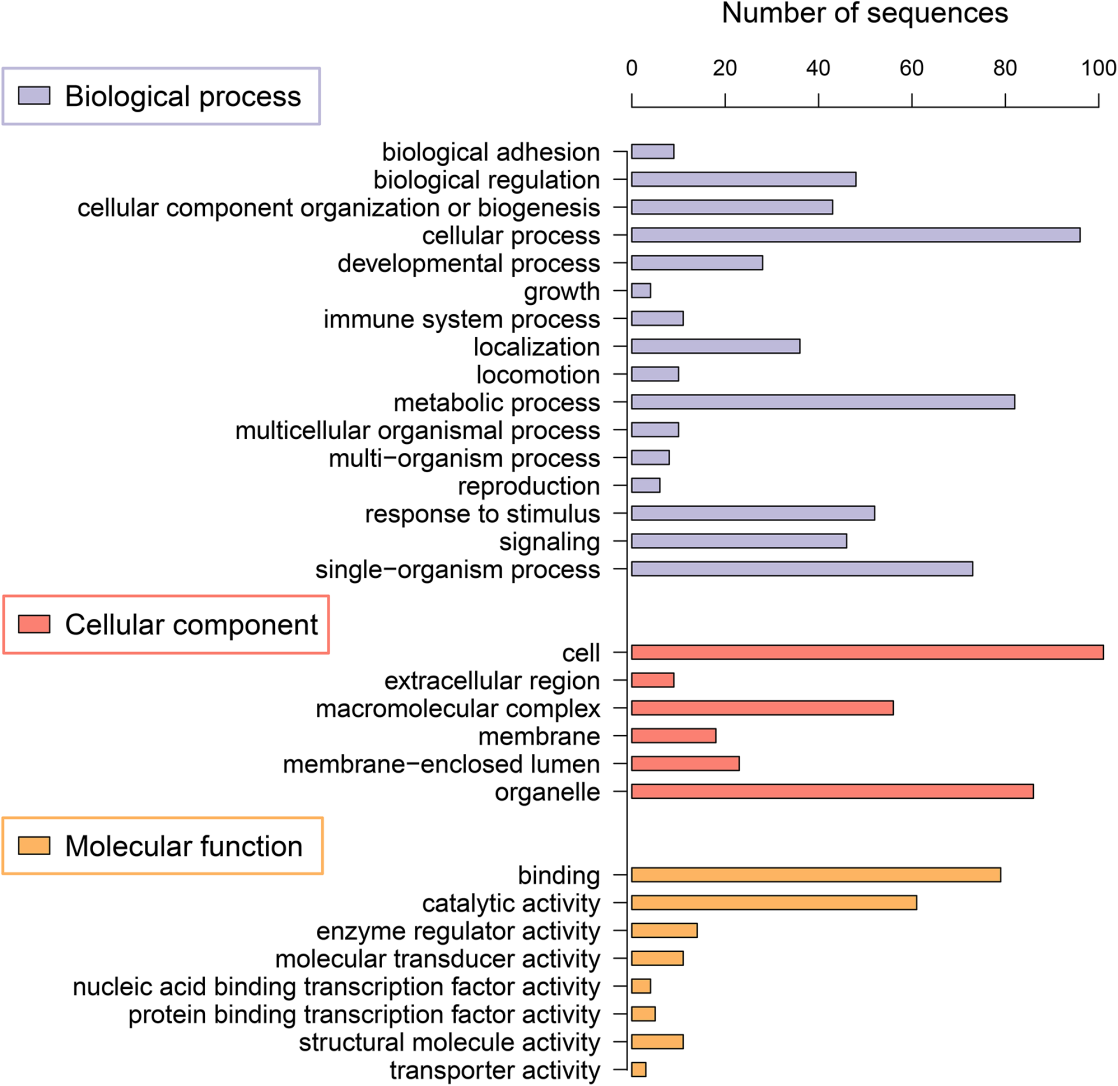
Histogram presentation of gene ontology (GO) term for putative coding transcripts. The GO terms were classified into different categories at level 2.

The list of all 253 coding transcripts arising from intergenic regions was analyzed using Blast2GO tools to provide insight into their potential biological functions. Out of these transcripts, 163 could be assigned at least one GO term, generating a total of 1,375 GO classifications (S4 Table). All these transcripts were grouped into 30 GO functional categories at level 2, which were distributed under the three main categories of biological process (BP, 16), molecular function (MF, 8), and cellular components (CC, 6) (Fig 4). Within the BP category, cellular process (17.08%) was the most dominant group, followed by metabolic process (14.59%) and single-organism process (12.99%). Two sub-categories of binding (42.02%) and catalytic activity (32.45%) were enriched in MF group. Regarding CC category, there were three highly represented clusters including cell (34.47%), organelle (29.35%) and macromolecular complex (19.11%) compared to other three sub-categories.

## Discussion

Recently, the increasing feed costs urge us to breed more efficient chickens through genetic improvement for profit maximization. Despite that several QTLs associated with RFI as a measure of feed efficiency have been identified, further refined exploration at the gene level is still required. To elucidate the genetic architecture underlying RFI, we provided a pioneering and comprehensive transcriptome profiling based on six chickens with extreme RFI performances. Our findings not only unearth many promising candidate genes implicated in RFI, but also gain new insight into their biological effects on feed efficiency. Evaluation of genetic merit based on functional genes would accelerate the genetic improvement of efficient chickens in the foreseeable future. In addition, assessing the global transcriptome landscape and annotating novel intergenic transcripts would assist in discovering new gene structures and improve current gene models.

The current RNA-Seq work provided greater sequence depth and obtained higher proportions of mapped reads than several previous chicken transcriptome studies ranging from 64.00 to 85.00% [40–43]. The high-quality sequences and superior mapping rates enabled the accuracy and reliability of further differential expression analysis. Despite that we enhanced the detection power of DEGs recommended by a previous study [28], the number (n = 41) of DEGs was still not high. The value is very close to a recent chicken RNA-Seq result (n = 40) [24], but is lower than another RNA-Seq experiment in chickens (n = 164) [40]. Firstly, the experimental population is a pure line of brown-egg dwarf layers with lower genetic variation at a global level. The similar genetic basis between two divergent conditions may cause concordant expression signals for most genes, and reduce the number of DEGs occurring at random [24]. Moreover, the number of DEGs was also greatly influenced by different detection algorithms and biological replicates [44–46]. Compared with QuasiSeq and DESeq used in the two aforementioned papers, DESeq2 (successor of DESeq) method provides greater inferential power in a typical RNA-Seq experiment with small replicate numbers [32, 45, 47].

Currently, the chicken QTL database deposited only 37 QTL regions associated with feed efficiency traits [34], and most of these QTLs suffered from wide confidence intervals covering dozens of genes or variants. This study is the first report for identification of functional determinants involved in RFI at the gene level by RNA-Seq in chickens. However, of particular interest is the poor concordance between DEGs and reported QTLs, which is in agreement with a previous study in chicken [48]. This outcome suggested that feed efficiency traits may be controlled by diverse QTLs or genes in different breeds, and pursuing the genetic evidence of feed efficiency by multiple methods and different populations is extremely essential.

To confirm the putative results from RNA-Seq, we randomly selected a subset of DEGs for qRT-PCR assays. Overall, there was excellent agreement and high concordance between the computational and experimental results, which was similar to some previous results in animals [23, 24, 40] and revealed good detection sensitivity and accuracy. After functional enrichment analyses, most GO terms and KEGG pathways were mainly involved in small molecule biosynthetic and metabolism processes. The results were also in accordance with several previous studies in cattle and pigs [6, 49, 50], and indicated that all identified DEGs may play important roles in controlling RFI through affecting digestive and metabolic processes [51]. It should be noted that negative genetic correlations between digestive efficiency and three feed efficiency traits (RFI, FI and FCR) were found [48], suggesting that the stronger digestive and metabolic abilities could lead to greater nutrient availability and compensate the lower feed intake in the more efficient chickens.

Generally, the difference in RFI performance between individual chickens attributes to five major biological processes including feed intake, digestibility and associated energy costs, metabolism and stress, physical activity and thermoregulation [51, 52], meaning that putative genes involved in these processes could be regarded as promising candidates associated with RFI. Considering that it is too redundant to discuss all genes and several genes do not have clear function in chickens, we only select five representative genes with potential functional evidence in feed efficiency.

As the most significant gene archived in the NCBI database, angiotensin I converting enzyme (peptidyl-dipeptidase A) 1 (*ACE*) has been reported to be a key element of the renin-angiotensin system (RAS) which can influence body energy homeostasis, fat accumulation and glucose tolerance [53, 54]. Particularly, *ACE* gene plays an important role in converting the inactive decapeptide angiotensin I (AngI) into the bioactive octapeptide angiotensin II (Ang II). Some previous results have demonstrated that infusion of Ang II could lead to reduced feed intake and body weight in rats [55–57]. In agreement with these studies, low RFI chickens consumed an average of 25 g less feed than their counterparts ranked as high RFI in the present work. In addition, another study revealed that homozygous *ACE* knockout mice had higher energy expenditure related to increased fatty acid metabolism in the liver compared with wild-type mice [58]. This result meant that less energy was used for growth and production in the same feed intake, which would result in higher RFI. Therefore, we speculated that the increased expression of *ACE* gene in the low RFI group may optimize the feed efficiency by reducing feed intake and/or energy expenditure.

Some biological pathways like lipid metabolism and cholesterol biosynthesis were identified to be associated with RFI [50, 59]. A previous study suggested that the gene encoding the radical S-adenosyl methionine domain containing 2 (*RSAD2*) could serve as a modulator of lipid content and affect the lipid to protein ratio in the liver [60]. The high expression level of *RSAD2* was always found in the tissue with the lower fat deposition. Additionally, some results supported that several body fat traits together with serum leptin concentration were positively related to RFI performance [51, 61]. The up-regulation of *RSAD2* in the low RFI group may lead to decreased feed intake, high energy utilization and few energy costs by modulating fatty acid and leptin metabolism. Furthermore, another two significantly differential genes, cytosolic calcium-dependent phospholipase A2, group IVA (*PLA2G4A*) and fatty acid hydroxylase domain containing 2 (*FAXDC2*), were suggested to be implicated in lipid metabolism, steroid biosynthesis and metabolic pathways [62, 63]. The expression alterations of the two genes may cause the difference in the digestive and metabolic abilities between the low and high RFI groups.

Oxidative stress response is also an important factor influencing RFI, because the procedure may be an energy-demanding process. Two previous studies indicated that high RFI individuals were susceptible to stress [64, 65]. As a member of the sestrin family, sestrin 3 gene (*SESN3*) is involved in the maintenance of physiological concentrations of reactive oxygen species, and participates in the oxidative stress pathway [66, 67]. Lower respond to environmental stressors may need fewer energy costs and show better feeding behavior, resulting higher feed efficiency. Overall, RFI performance is a complex physiological process and variation in RFI may represent numerous intrinsic factors. Although we have identified 41 promising candidate genes, further investigation by increasing sample size and integrating different algorithms is critical to elucidate the biological mechanisms behind RFI.

It should be noted that an average of 18.88% matched reads were mapped to intergenic areas, suggesting that the current gene annotation in the chicken genome still needs to be further improved to determine the structures and functions of novel genes [68]. During transcript assembly and coding potential prediction, we employed stringent quality management to exclude likely false positives, resulting in fewer transcripts compared with a previous study [42]. In fact, the 38 putative non-coding transcripts could be regarded as long intergenic non-coding RNA (lincRNA) based on our quality control procedure. The fewer lincRNAs may be due to the fact that our RNA-Seq libraries are based on poly(A)+ mRNAs selection protocol. In this sense, only the lincRNAs with poly(A) tails could be identified while a number of transcripts are known to lack a classical poly(A) tail [69]. Hence, to detect and characterize all lncRNAs in detail, the specific library preparation procedure with rRNA depletion to enrich for non-rRNAs must be required [70]. Most protein-coding transcripts were located in unknown genomic contigs, suggesting that these genomic sequences may contain more novel genes and need further annotation [68, 71]. The Blast2GO results demonstrated that a majority of coding transcripts were responsible for fundamental biological regulation and metabolism processes.

## Conclusions

In summary, we conducted a comprehensive differential expression analysis and characterized global trancriptome architectures based on high-quality RNA-Seq data, and subsequently performed functional annotation for these putative associated genes and protein-coding transcripts. We identified a total of 41 differentially expressed genes associated with RFI. These promising genes play a critical role in digestibility, metabolism, stress response and energy homeostasis, hence resulting in divergent RFI performances. Among 10 randomly chosen genes, nine were successfully validated. We also discovered 253 intergenic coding transcripts, which may be from some unannotated genes. Our findings lay the foundation for comprehensive understanding of RFI, are beneficial to direct future breeding schemes improving feed efficiency and assist in optimizing the current gene models.

## Supporting Information

S1 Table Primers information and validation results of the 10 chosen differentially expressed genes by qRT-PCR analysis. (DOCX)

S2 Table GO enrichment analysis of 41 differentially expressed genes associated with residual feed intake. (DOCX)

S3 Table Summary of the KEGG analysis of 41 differentially expressed genes associated with residual feed intake. (DOCX)

S4 Table Detailed Blast2GO information for putative coding transcripts. (XLSX)
