# Supplementary material for: In-Depth Duodenal Transcriptome Survey in Chickens with Divergent Feed Efficiency Using RNA-Seq
Source: PLoS One. 2015 Sep 29;10(9):e0136765. doi: 10.1371/journal.pone.0136765 (PMC4721924; doi:10.1371/journal.pone.0136765)
Supplement: S1 Table — (DOCX) [file pone.0136765.s001.docx]

**S1 Table. Primers information and validation results of the 10 chosen differentially expressed genes by qRT-PCR analysis**

| **Ensembl gene ID^a^** | | **Log2 FC by RNA-Seq** | **Log2 FC by qRT-PCR** | **Forward primer sequence** | **Reverse primer Sequence** | **Amplicon (bp)** |
| --- | --- | --- | --- | --- | --- | --- |
| ENSGALG00000013738 | | 0.72 | -0.55 | GTGGAGATTGGGATGAACG | CTCTTCTTCCTGGTTAGTTTGC | 297 |
| ENSGALG00000021040 | | 1.02 | 0.51 | GCATTGGCACTGGCATCT | CCATCCCTTCCCCACTCAT | 134 |
| ENSGALG00000021262 | | 0.66 | 0.46 | GGGATAGGCTTTGGCTTCG | CGCATTGATGGGGTCGTA | 210 |
| ENSGALG00000009560 | | 0.63 | 0.54 | CATCCATAAGGTTCACCACGAG | AAAGGCACCAAATGAAGAGG | 223 |
| ENSGALG00000014281 | | -1.22 | -0.64 | GCAGCCACCTGTTTGTCA | TTGGGTTATCCTCCAGTATCAT | 128 |
| ENSGALG00000003972 | | 0.92 | 0.82 | AATCGCAGCGTGGTTCTC | CTCGTAGGCTTTGCTTTGTC | 193 |
| ENSGALG00000005453 | | -0.96 | -0.61 | TATGACTACAGAGCCAAACGAA | CATAATCCCCTTTCCACCAG | 103 |
| ENSGALG00000011805 | | 0.73 | 0.54 | AGACGATGGGAAAAGCAGA | GCAGTGAAGTCCTCCAACAA | 104 |
| ENSGALG00000017283 | | -1.15 | -0.84 | CCATCAAACCCCAAGAGC | GTGGCACAAAGGGCAATG | 181 |
| ENSGALG00000020679 | | 0.81 | 0.30 | GCAAGAGTTGGATATTTTGGTC | AACATCCCAGTCGGCAAGG | 116 |
| ENSGALG00000014442 (*GAPDH* as control) | -0.02 | | 0.00 | CGTCCTCTCTGGCAAAGTCC | TTCCCGTTCTCAGCCTTGAC | 132 |

Abbreviations: FC = fold change.

^a^Identification of the gene according to Ensembl genes database 76.
