# Supplementary material for: In-Depth Duodenal Transcriptome Survey in Chickens with Divergent Feed Efficiency Using RNA-Seq
Source: PLoS One. 2015 Sep 29;10(9):e0136765. doi: 10.1371/journal.pone.0136765 (PMC4721924; doi:10.1371/journal.pone.0136765)
Supplement: S2 Table — (DOCX) [file pone.0136765.s002.docx]

**S2 Table. GO enrichment analysis of 41 differentially expressed genes associated with residual feed intake**

| **Accession no.** | **Ontology** | **Definition** | **No. of DEGs** | ***P*-value** |
| --- | --- | --- | --- | --- |
| GO:0016053 | BP | organic acid biosynthetic process | 3 | 0.000242 |
| GO:0046394 | BP | carboxylic acid biosynthetic process | 3 | 0.000242 |
| GO:0044283 | BP | small molecule biosynthetic process | 3 | 0.000815 |
| GO:0019752 | BP | carboxylic acid metabolic process | 3 | 0.003515 |
| GO:0043436 | BP | oxoacid metabolic process | 3 | 0.003728 |
| GO:0006082 | BP | organic acid metabolic process | 3 | 0.004126 |
| GO:0060541 | BP | respiratory system development | 2 | 0.005109 |
| GO:0044711 | BP | single-organism biosynthetic process | 3 | 0.01212 |
| GO:0030855 | BP | epithelial cell differentiation | 2 | 0.017628 |
| GO:0044255 | BP | cellular lipid metabolic process | 2 | 0.018297 |
| GO:0035295 | BP | tube development | 2 | 0.021412 |
| GO:0012505 | CC | endomembrane system | 4 | 0.022631 |
| GO:0005488 | MF | binding | 8 | 0.02768 |
| GO:0007417 | BP | central nervous system development | 2 | 0.034935 |
| GO:0016788 | MF | hydrolase activity, acting on ester bonds | 2 | 0.03986 |
| GO:0006629 | BP | lipid metabolic process | 2 | 0.040542 |
| GO:0000904 | BP | cell morphogenesis involved in differentiation | 2 | 0.045539 |

Abbreviations: GO = gene ontology; BP = biological process; MF = molecular function; CC = cellular components; DEG = differentially expressed gene.
