# Supplementary material for: In-Depth Duodenal Transcriptome Survey in Chickens with Divergent Feed Efficiency Using RNA-Seq
Source: PLoS One. 2015 Sep 29;10(9):e0136765. doi: 10.1371/journal.pone.0136765 (PMC4721924; doi:10.1371/journal.pone.0136765)
Supplement: S3 Table — (DOCX) [file pone.0136765.s003.docx]

**S3 Table. Summary of the KEGG analysis of 41 differentially expressed genes associated with residual feed intake**

| **Accession no.** | **Definition** | **No. of DEGs** | ***P*-value** |
| --- | --- | --- | --- |
| map00100 | Steroid biosynthesis | 2 | 0.001154141 |
| map04115 | p53 signaling pathway | 2 | 0.012325764 |
| map00564 | Glycerophospholipid metabolism | 2 | 0.017419664 |
| map04370 | VEGF signaling pathway | 2 | 0.018476838 |
| map04070 | Phosphatidylinositol signaling system | 2 | 0.033330302 |
| map01100 | Metabolic pathways | 6 | 0.040059886 |

Abbreviations: KEGG = kyoto encyclopedia of genes and genomes; DEG = differentially expressed gene.
